# Supplementary material for: Park Visitors and Birds Connected by Trade-Offs and Synergies of Ecosystem Services
Source: Animals (Basel). 2025 Sep 6;15(17):2619. doi: 10.3390/ani15172619 (PMC12427294; doi:10.3390/ani15172619)
Supplement: Supplementary file 1 [file animals-15-02619-s001.zip › animals-3799142-supplementary.pdf]

S1: Contents of the PPGIS - survey "A survey on visitors' use of park cultural services and attitudes towards birds in Xingqing Palace Park". We conducted the survey using electronic questionnaires coupled with tablet-based mapping.

A survey on visitors' use of park cultural services and attitudes towards birds in Xingqing Palace Park

This questionnaire was used to survey residents and visitors about their use of cultural services and their attitudes of birds in Xingqing Palace Park, and the results of the survey were used for research purposes only and are completely anonymous and non-private.

☐ I agree with the statements above and I am willing to participate in the survey
☐ No, I don't agree.

The following questions about your experience in the park

1. I can better foster relationships with others in this park. [Single Choice]

☐ Strongly disagree
☐ Disagree
☐ Neutrality
☐ Agree
☐ Strongly agree

2. I may play sports, take a walk, or engage in other activities in this park. [Single Choice]

☐ Strongly disagree
☐ Disagree
☐ Neutrality
☐ Agree
☐ Strongly agree

3. There is a lot of beautiful scenery that can cheer me up in this park. [Single Choice]

☐ Strongly disagree
☐ Disagree
☐ Neutrality
☐ Agree
☐ Strongly agree

4. I can learn much about history, wildlife, flora, and cultural customs in this park. [Single Choice]

☐ Strongly disagree
☐ Disagree

☐ Neutrality
☐ Agree
☐ Strongly agree

5. I can locate historical and cultural heritage, and sense its history and culture in this park. [Single Choice]

☐ Strongly disagree
☐ Disagree
☐ Neutrality
☐ Agree
☐ Strongly agree

6. I frequently get inspired to think of new ideas for work, life, or studies in this park. [Single Choice]

☐ Strongly disagree
☐ Disagree
☐ Neutrality
☐ Agree
☐ Strongly agree

7. Because this park reminds me of many intriguing things or pleasant memories I have had here, I frequently feel like I belong there. [Single Choice]

☐ Strongly disagree
☐ Disagree
☐ Neutrality
☐ Agree
☐ Strongly agree

The following questions about your attitudes toward birds in the park

8. I am more likely to be attracted to the pleasant, melodious sounds of birds in this park. [Single Choice]

☐ Strongly disagree
☐ Disagree
☐ Neutrality
☐ Agree
☐ Strongly agree

9. I am more likely to be attracted to the bright and colorful plumage of birds in this park. [Single Choice]

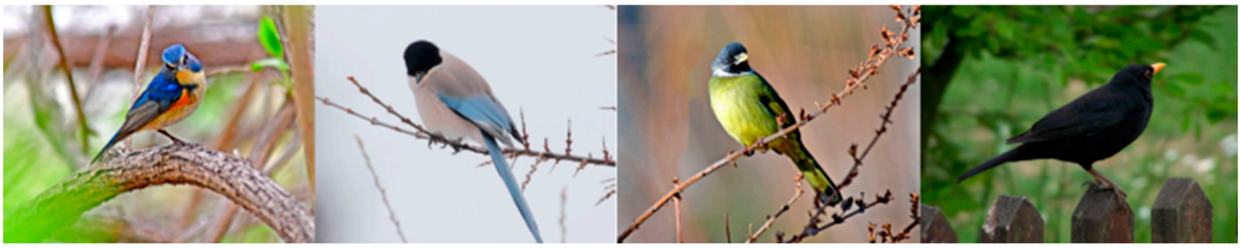

☐ Strongly disagree
☐ Disagree
☐ Neutrality
☐ Agree
☐ Strongly agree

10. I am more likely to be attracted by the long or short tails and large or small sizes of the birds in this park. [Single Choice]

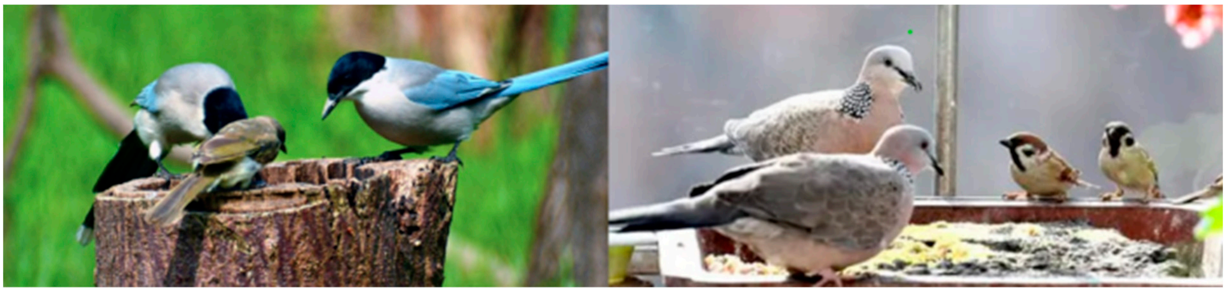

☐ Strongly disagree
☐ Disagree
☐ Neutrality
☐ Agree
☐ Strongly agree

11. I am more attracted to the behavior of the birds in the park as they feed, play, and fly freely. [Single Choice]

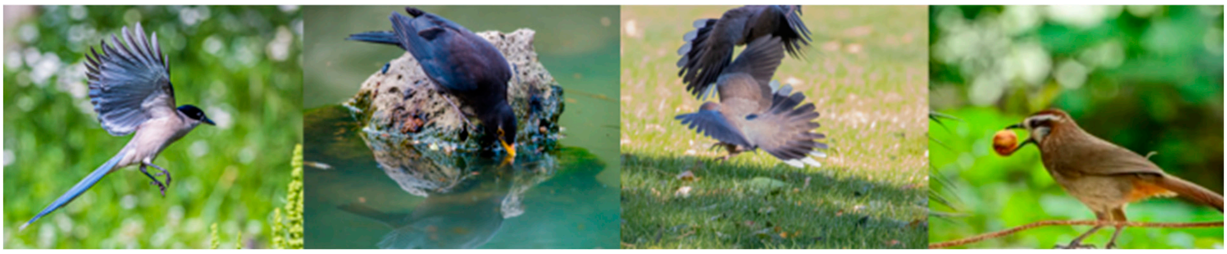

☐ Strongly disagree
☐ Disagree
☐ Neutrality
☐ Agree
☐ Strongly agree

Socio-demographic characteristics

12. What is your gender? [Single Choice]

☐ Female
☐ Male
☐ Other

13. How old are you? [Single Choice]

☐ 14 years old and below
☐ 15 - 34 years old
☐ 35 - 59 years old
☐ Over 60 years old

14. What is your level of education? [Single Choice]

☐ Junior high school or below
☐ High school (vocational school, junior college technical school, etc.)
☐ Bachelor's degree
☐ Master's degree or above

15. What is your average monthly income? [Single Choice]

☐ Under ￥2000
☐ ￥2000 - ￥4000
☐ ￥4000 - ￥6000
☐ More than ￥6000

16. Please rank your visiting purpose of this park (choose 4 items and rank them in descending order of importance). [Ranking Choice]

☐ Exercise
☐ Picnic
☐ Chatting and relaxing
☐ Spending time with family
☐ Relaxation
☐ Traveling and take photos
☐ Babysitting
☐ Outdoor Learning
☐ Getting close to nature

17. How often do you visit the park? [Single Choice]

☐ 1-3 times per week
☐ 1-3 times per month
☐ 1-3 times per half year
☐ Once a year or less

The Allocation and labeling of cultural ecosystem service social values

18. Allocate the hypothetical 100 RMB to the following 7 categories of services following your perceived importance. [fill in the blank]

Social relations value

Recreational value

Aesthetic value

Educational value

Cultural heritage value

Inspiration

Sense of place

19. Mark the locations on the map where you believe the services that you allocated are available.

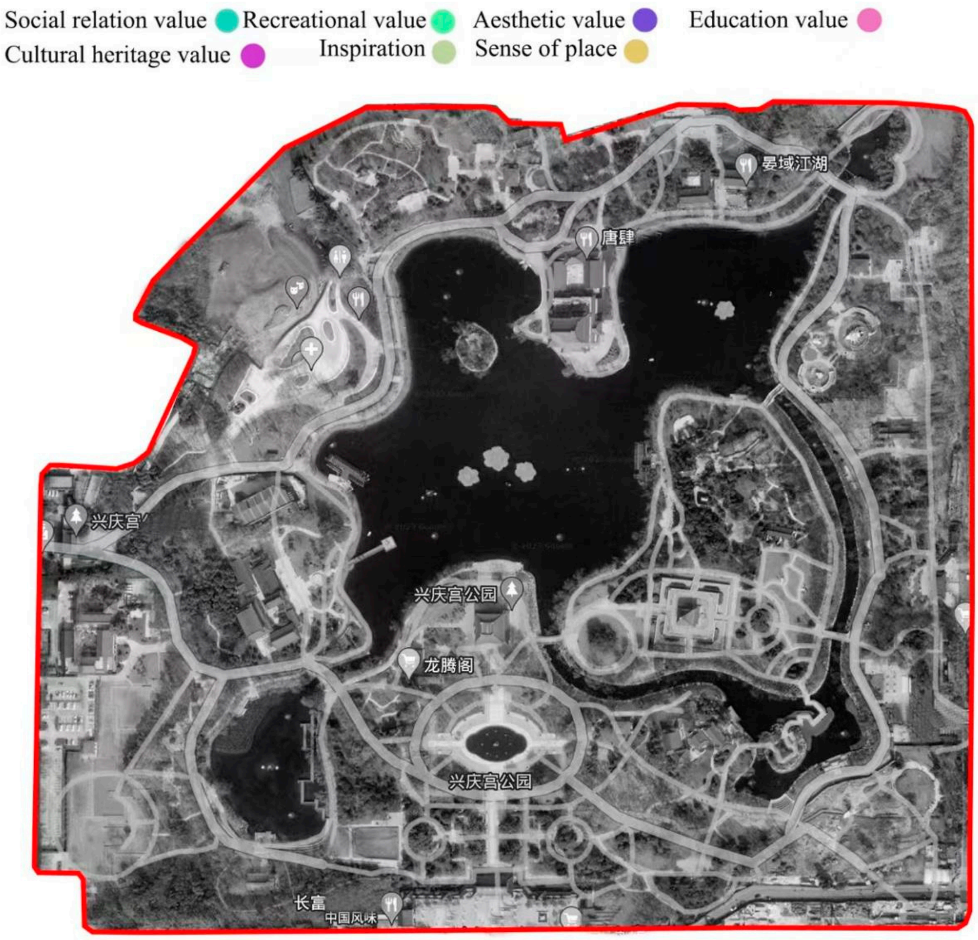

S2: Species list in the bird census of Xingqing Palace Park.

Species	Latin name	Abudance	Dominance	Feeding guilds
Azure-winged Magpie	*Cyanopica cyanus*	169	22.7%	Invertivore
Tree Sparrow	*Passer montanus*	169	22.7%	Granivore
White-browed Laughingthrush	*Garrulax sannio*	78	10.5%	Invertivore
Light-vented Bulbul	*Pycnonotus sinensis*	69	9.3%	Omnivore
Barn Swallow	*Hirundo rustica*	49	6.6%	Invertivore
Spotted Dove	*Spilopelia chinensis*	45	6.0%	Granivore
White-cheeked Starling	*Spodiopsar cineraceus*	45	6.0%	Invertivore
Crested Myna	*Acridotheres cristatellus*	32	4.3%	Omnivore
Common Swift	*Apus apus*	15	2.0%	Invertivore
Oriental Turtle-dove	*Streptopelia orientalis*	12	1.6%	Granivore
Yellow-billed Grosbeak	*Eophona migratoria*	12	1.6%	Omnivore
Eurasian Blackbird	*Turdus merula*	9	1.2%	Omnivore

Black-crowned Night-heron	*Nycticorax nycticorax*	6	0.8%	Piscivore
Little Grebe	*Tachybaptus ruficollis*	6	0.8%	Invertivore
Common Tern	*Sterna hirundo*	6	0.8%	Piscivore
Brown Shrike	*Lanius cristatus*	6	0.8%	Invertivore
Great Cormorant	*Phalacrocorax carbo*	5	0.7%	Piscivore
Black-throated Tit	*Aegithalos concinnus*	3	0.4%	Invertivore
Eurasian Magpie	*Pica pica*	2	0.3%	Granivore
Oriental Greenfinch	*Chloris sinica*	2	0.3%	Granivore
Japanese Thrush	*Turdus cardis*	1	0.1%	Omnivore
Asian Brown Flycatcher	*Muscicapa dauurica*	1	0.1%	Invertivore
European Kingfisher	*Alcedo atthis*	1	0.1%	Piscivore
Long-tailed Tit	*Aegithalos glaucogularis*	1	0.1%	Invertivore
Yellow-browed Willow Warbler	*Phylloscopus inornatus*	1	0.1%	Invertivore

S3: The AUC for various bird species from operating the MaxEnt model.

species	Latin name	AUC
Azure-winged Magpie	*Cyanopica cyanus*	0.743
Tree Sparrow	*Passer montanus*	0.683
White-browed Laughingthrush	*Garrulax sannio*	0.729
Light-vented Bulbul	*Pycnonotus sinensis*	0.754
Swallow	*Hirundo rustica*	0.858
Spotted Dove	*Spilopelia chinensis*	0.665
White-cheeked Starling	*Spodiopsar cineraceus*	0.808
Crested Myna	*Acridotheres cristatellus*	0.699
Common Swift	*Apus apus*	*[Null]
Oriental Turtle-dove	*Streptopelia orientalis*	0.756
Yellow-billed Grosbeak	*Eophona migratoria*	0.708
Eurasian Blackbird	*Turdus merula*	0.664
Black-crowned Night-heron	*Nycticorax nycticorax*	[Null]
Little Grebe	*Tachybaptus ruficollis*	0.873
Common Tern	*Sterna hirundo*	0.909
Brown Shrike	*Lanius cristatus*	0.895
Great Cormorant	*Phalacrocorax carbo*	[Null]
Black-throated Tit	*Aegithalos concinnus*	[Null]
Eurasian Magpie	*Pica pica*	[Null]
Oriental Greenfinch	*Chloris sinica*	[Null]

Japanese Thrush	*Turdus cardis*	[Null]
Asian Brown Flycatcher	*Muscicapa dauurica*	[Null]
European Kingfisher	*Alcedo atthis*	[Null]
Long-tailed Tit	*Aegithalos glaucogularis*	[Null]
Yellow-browed Willow Warbler	*Phylloscopus inornatus*	[Null]

*Insufficient points to calculate AUC.

S4: The comparison of socio-demographic characteristics of respondents.

Socio-demographic category		Questionnaire results (%)	*Percentage in Xi'an (%)
Genders	Male	49.08	51.07
	Female	50.92	48.03
Ages(years)	Below 14	6.75	15.65
	15-34	37.42	68.33
	35-59	32.52	
	Above 60	23.31	16.02
Qualifications	Junior high school and below	12.27	46.02
	High school (vocational school, junior college technical school, etc.)	25.77	20.22
	Bachelor's degree	49.69	33.76
	Master or above	12.27	
Monthly income	Less than ￥2000	16.56	
	￥2000-￥4000	19.63	No data
	￥4000-￥6000	28.83	
	Above ￥6000	34.98	

*The primary data from Xi'an's 7th National Census (Xi'an Municipal Bureau of Statistics, 2021)
